# Toll-like receptors in infectious myocarditis: pathogen-specific recognition, spatiotemporal dynamic regulation and clinical translation

**DOI:** 10.3389/fcvm.2026.1881939

**Published:** 2026-07-15

**Authors:** Yuhua Li, Weiming Liao, Qingfei Liang, Yanhua Li

**Affiliations:** 1 Department of Pharmacy, The First Naval Hospital of Southern Theatre Command, Zhanjiang, Guangdong, China; 2 Department of Cardiology, Air Force Hospital of PLA Southern Theater Command, Guangzhou, China; 3 School of Basic Medical Sciences, Guangdong Medical University, Zhanjiang, Guangdong, China; 4 Department of Cardiovascular Medicine, Chinese PLA General Hospital & Chinese PLA Medical School, Beijing, China

**Keywords:** clinical translation, infectious myocarditis, pathogen-specific recognition, spatiotemporal dynamic regulation, targeted intervention, toll-Like receptors

## Abstract

Infectious myocarditis is a life-threatening cardiovascular inflammatory disorder characterized by high heterogeneity in clinical onset, progression and prognosis. Large-sample clinical data have demonstrated that the in-hospital mortality of COVID-19-associated myocarditis reaches 19.4%, significantly higher than that of influenza-associated myocarditis (10.5%). Additionally, the incidence of adeno-associated virus (AAV) gene therapy-related myocarditis is 6.2%, while the mortality of sepsis-associated myocarditis is as high as 70%–90%. Toll-like receptors (TLRs), the core pattern recognition receptors of innate immunity, dominate the entire pathological cascade, ranging from pathogen recognition and acute inflammatory burst to myocardial injury and chronic fibrous remodeling. Nevertheless, most current studies merely focus on the linear correlation between individual TLR activation and myocardial inflammation, failing to systematically clarify pathogen-TLR matching specificity and the spatiotemporal dynamic regulatory mechanisms of TLR signaling throughout disease progression. This review comprehensively combs the latest epidemiological profiles of infectious myocarditis, characterizes the expression patterns and signaling regulatory features of the TLR family within the cardiac immune microenvironment, analyzes pathogen-specific recognition modes mediated by common pathogens, elaborates the spatiotemporal regulatory rules of TLR signaling across acute inflammation, immune deviation and chronic fibrosis stages, and summarizes pathogen-oriented intervention strategies as well as relevant translational bottlenecks. Cumulative clinical evidence confirms that pathogen-TLR matching determines inflammatory phenotypes and severity of infectious myocarditis, and that the spatiotemporal dynamics of TLR signaling directly govern disease progression. Notably, TLR-targeted therapies must adhere to the core principles of pathogen specificity and staged precise regulation. This review provides a systematic theoretical basis for precise immunodiagnosis and individualized immunotherapy of infectious myocarditis.

## 1 Introduction

Infectious myocarditis is a prevalent yet severe inflammatory cardiovascular condition triggered by the invasion of exogenous pathogenic microorganisms and sustained activation of endogenous danger signals. Aberrant activation and dysregulated innate immune signaling are recognized as the core pathological mechanisms driving disease onset and progression. As pivotal pattern recognition receptors capable of specifically identifying pathogen-associated molecular patterns and damage-associated molecular patterns, TLRs are extensively involved in the entire pathological cascade: from initial pathogen recognition and inflammatory signal initiation, to cardiomyocyte structural damage and massive immune cell infiltration, and ultimately to late chronic fibrous remodeling. TLRs thus serve as the critical hub for deciphering the complex pathogenesis of infectious myocarditis and exploring viable precise intervention targets. Current research is largely confined to simple linear correlations between individual TLR activation and myocardial inflammatory injury. Existing studies neither systematically explain the specificity of TLR subtype matching driven by varying pathogen spectra, nor clarify the spatiotemporal dynamic features of TLR signaling across the acute, transitional and chronic phases of the disease. The joint mechanism by which pathogen-TLR specific binding and TLR spatiotemporal dynamics mediate myocarditis progression remains poorly elucidated, which also forms the core rationale for this systematic review based on available high-quality evidence (1, 2).

### 1.1 Epidemiological characteristics of infectious myocarditis

This section systematically summarizes the epidemiological characteristics of infectious myocarditis caused by exogenous pathogenic microorganisms. Of note, abnormal activation of endogenous retroviruses (ERVs) and myocardial injury associated with adeno-associated virus (AAV) gene therapy vectors share the innate immune mechanism of TLR-mediated nucleic acid recognition with infectious myocarditis, and belong to the category of infection-like innate immune activation. Immune checkpoint inhibitor (ICI)-associated myocarditis is classified as non-infectious myocardial injury related to immunotherapy, which is only referred to for mechanistic analogy in this review and is not included in the core scope of infectious myocarditis.

In recent years, alongside shifts in global epidemiological trends and advances in clinical medical technology, the pathogen spectrum and epidemiological profiles of infectious myocarditis have undergone marked changes. Although viruses remain the primary causative agents, the proportion of secondary bacterial infections, opportunistic fungal infections in immunocompromised hosts, and iatrogenic immune-related myocardial injury has risen steadily year by year. Myocarditis of different etiologies exhibits substantial heterogeneity in onset risk, susceptible populations and clinical prognosis, posing considerable challenges to clinical diagnosis and targeted therapy.

#### 1.1.1 Viral pathogens

The global COVID-19 pandemic has markedly elevated the overall population risk of myocarditis and pericarditis. Relevant studies indicate that individuals infected with COVID-19 carry a 15.7-fold higher risk of myocarditis than uninfected populations. The incidence of chronic myocarditis following COVID-19 infection ranges from 0.4% to 28.9%, with onset disparities closely associated with patients' underlying comorbidities and age (3, 4). A total of 27,725 adult hospitalized myocarditis cases were recorded across the United States from 2019 to 2020, among which 21.1% were directly linked to COVID-19 infection and 3.7% to influenza virus infection. The total hospitalization volume increased approximately 1.5 times compared with the pre-pandemic period. Furthermore, in-hospital mortality associated with COVID-19-related myocarditis is notably higher than that of influenza-related myocarditis, implying that SARS-CoV-2-mediated myocardial injury carries greater lethality and destructive potential (5).

Emerging arbovirus infections have become an increasingly non-negligible cause of viral myocarditis in recent years. The incidence of cardiac events among dengue fever patients reaches as high as 27.21%, with myocarditis standing out as the most common cardiac complication of dengue infection (6). Severe yellow fever is associated with an extremely high rate of myocardial injury, and 12.3% of fatal cases are pathologically confirmed as viral myocarditis, representing a leading cause of yellow fever-related mortality (7).

Within the traditional viral pathogen spectrum, coxsackievirus group B remains the predominant pathogen of viral myocarditis in adolescents, and also acts as a key contributor to sudden cardiac death and progression to dilated cardiomyopathy in this population (8). Enterovirus D68 reached an epidemic peak in Europe during 2021–2022. Infection with this virus can directly induce severe cardiovascular complications, further broadening the pathogen spectrum of viral myocarditis (9).

In addition, 30%–40% of clinical myocarditis cases fail to identify a clear pathogen and are classified as idiopathic myocarditis. Up to 30% of patients with biopsy-confirmed myocarditis eventually progress to dilated cardiomyopathy. Chronic disease progression and end-stage heart failure represent the most critical long-term adverse prognostic outcomes of myocarditis (10).

#### 1.1.2 Non-viral infectious pathogens

Among myocardial injuries induced by non-viral infections, septic cardiomyopathy primarily affects the heart, with lipopolysaccharide translocation resulting from intestinal flora imbalance serving as its core pathogenic trigger. Sepsis has evolved into a major global public health burden, causing over 11 million annual deaths worldwide in recent years. The mortality rate of sepsis-related myocardial dysfunction remains as high as 70%–90% (11, 12). Gram-negative bacteria are the dominant pathogens underlying sepsis, with *Escherichia coli* being the most prevalent clinical strain. Myocardial inflammation mediated by this pathogen is characterized by acute onset and severe tissue injury (13).

#### 1.1.3 Iatrogenic immune-mediated myocardial injury

Among iatrogenic myocardial injuries, immune checkpoint inhibitor (ICI)-associated myocarditis represents the most lethal drug-induced cardiotoxicity in clinical practice. Combined therapeutic regimens further elevate myocarditis risk, becoming a major factor restricting the clinical application of tumor immunotherapy (14). The overall incidence of AAV gene therapy-related myocardial injury or myocarditis is 6.2%, with onset peaking in the second week after vector infusion. Such adverse events occur exclusively in patients with muscular or cardiac diseases, showing distinct population specificity and temporal window features (15).

Moreover, patients with COVID-19 complicated by underlying cardiovascular conditions face a markedly elevated risk of adverse cardiovascular sequelae including myocarditis (16). Sepsis survivors also experience a significant increase in long-term cardiovascular adverse events due to persistent myocardial dysfunction (17).

The pronounced heterogeneity in onset patterns and clinical outcomes of myocarditis induced by diverse pathogens and clinical scenarios implies underlying disparities in pathogen-specific immune regulatory mechanisms, laying a key clinical foundation for this review to explore pathogen-specific recognition and spatiotemporal dynamic regulation of TLRs.

### 1.2 TLR family and distribution in cardiac immune microenvironment

Building on the epidemiological heterogeneity of infectious myocarditis, further dissection of the classification, signaling pathways and expression distribution of the TLR family in the cardiac immune microenvironment is a prerequisite for understanding pathogen-specific recognition and spatiotemporal dynamic regulation.

#### 1.2.1 Classification and signaling pathways of TLR family

Based on subcellular localization, the TLR family is categorized into two subgroups. Membrane-bound TLRs (TLR1/2/4/6) primarily recognize extracellular microbial membrane structural components, while intracellular TLRs (TLR3/7/8/9) target intracellular pathogenic constituents such as viral nucleic acids. The intensity and duration of TLR signaling activation are precisely modulated rather than unrestrainedly amplified, being collectively governed by positive and negative regulatory networks. Multiple accessory molecules exert indispensable roles in fine-tuning TLR-mediated immune responses (18, 19).

#### 1.2.2 Cell-specific expression of TLRs in cardiac immune microenvironment

TLRs are predominantly expressed in leukocytes, but are also present in parenchymal cells including cardiomyocytes, fibroblasts and endothelial cells. Among TLR subtypes, TLR4, TLR2, TLR3 and TLR5 exhibit the highest expression levels in the heart, among which TLR4 and TLR2 have been most extensively investigated in the pathological process of myocardial injury. TLR4 is constitutively expressed in adult and failing cardiomyocytes, and its expression level in cardiac tissue is further elevated after myocardial infarction (20).

TLR4 is the most abundantly expressed TLR subtype within the cardiac immune microenvironment. Core cardiac cell types including cardiomyocytes, macrophages and fibroblasts constitutively express TLRs with clear functional differentiation. Cardiomyocytes predominantly express TLR3/4/9 and undertake the initial sensing of pathogenic and damage-associated signals. Macrophages highly express TLR2/4/7/9 and serve as core effector cells for inflammatory signal amplification. Fibroblasts mainly express TLR2/4 and participate in chronic inflammation and fibrotic progression (21, 22).

Single-cell RNA sequencing of the human fetal heart has revealed that TLR2 is highly expressed in cardiac resident macrophages, while molecules related to the TLR signaling pathway are also detected at low levels in non-immune cells such as cardiomyocytes, fibroblasts and endocardial endothelial cells, presenting cell type-specific expression profiles (23).

Neonatal rat cardiomyocytes show constitutive expression of TLR2. Mechanical stretch simulating pressure overload can upregulate TLR2 expression in cardiomyocytes, indicating that TLR2 is not only expressed in immune cells, but also functionally expressed in cardiomyocytes *per se* and participates in injury response (24).

In mouse ventricular myocardium after myocardial infarction and in primary cardiomyocytes subjected to hypoxia, both protein and mRNA expression levels of TLR4 are significantly upregulated (25). Elevated TLR4 expression has also been detected in myocardial tissue of mice with acute myocardial infarction and in hypoxia-challenged cardiomyocytes, and TLR4 serves as a therapeutic target of relevant cardioprotective agents (26).

*In vivo* two-photon microscopy imaging of the spatiotemporal evolution of cardiac inflammation after myocardial infarction has demonstrated upregulated TLR4 expression in ischemic myocardial tissue and infiltrating immune cells. TLR4 expression is detectable in both cardiomyocytes and infiltrating leukocytes, providing visual evidence for the *in vivo* distribution of TLR4 and its upregulation upon injury (27).

Among infiltrating immune cells, plasmacytoid dendritic cells (pDCs) exhibit unique TLR expression profiles. This cell subtype abundantly expresses intracellular TLR7/9, acting as a key immune cell for recognizing viral nucleic acids and initiating antiviral innate immune responses (28).

Upon activation, TLR signaling initiates and propagates inflammatory cascades via downstream pathways such as MAPKs and NF-*κ*B in cardiomyocytes, directly mediating the progression of myocardial inflammatory injury (29). The magnitude of TLR signaling activation is also precisely constrained by endogenous negative regulators including SIGIRR and miRNAs to maintain cardiac immune homeostasis (30, 31). Multiple preclinical and clinical studies have verified that inhibition of the TLR4 signaling cascade can markedly alleviate myocardial inflammatory damage in viral myocarditis, providing solid theoretical evidence for TLR4 as a core intervention target for infectious myocarditis (32). Notably, current evidence on TLR distribution in the heart is predominantly derived from cell-type-specific expression profiling. Studies delineating TLR expression across anatomical subregions of the heart (e.g., pericardium, myocardium, endocardium, apical/basal segments, and ventricular walls) remain scarce, and high-resolution spatial transcriptomic evidence at the anatomical level is still lacking. This represents an important direction for future investigation with the advancement of spatial omics technologies.

#### 1.2.3 Negative regulatory network of TLR signaling

As an essential member of the TIR family, SIGIRR negatively modulates TLR4-mediated inflammatory signaling, thereby mitigating cardiomyocyte and endothelial cell injury triggered by excessive inflammatory responses (30). As post-transcriptional regulatory molecules, miRNAs directly participate in the negative modulation of TLR signaling pathways, further fine-tuning the activation intensity of TLR cascades and sustaining homeostasis within the cardiac immune microenvironment (31).

### 1.3 Purpose and structure of this review

Based on the above basic characteristics of the TLR family and current research limitations, this review is systematically organized around the core logical axis of pathogen-TLR matching.

Most existing reviews adopt a simplistic linear analytical framework of “TLR activation → inflammation → myocardial injury”, while neglecting subtype disparities in TLR recognition induced by different pathogens and the spatiotemporal dynamic evolution of TLR signaling across myocarditis stages. This limitation leads to a disconnection between research conclusions and clinical practice (1, 33).

Centered on the core logical axis of pathogen-TLR matching, this review systematically integrates TLR recognition mechanisms mediated by various inducing factors, including viruses, bacteria, fungi, endogenous retroviruses and AAV-related iatrogenic gene therapy vectors. It further analyzes the dynamic regulatory patterns of TLR signaling across three pivotal stages: acute inflammatory flare, immune tolerance breakdown and autoimmune transformation, as well as chronic fibrotic remodeling. Additionally, this work comprehensively summarizes pathogen-oriented precise TLR-targeted intervention strategies and relevant clinical translational bottlenecks. Based on high-quality published evidence, this review systematically clarifies that pathogen-TLR matching directly determines the inflammatory phenotype of infectious myocarditis, and spatiotemporal dynamic alterations in TLR signaling act as the core regulatory factor governing myocarditis progression (2).

## 2 Pathogen-specific TLR recognition in infectious myocarditis

Different pathogens selectively activate specific TLR subtypes via their unique pathogen-associated molecular patterns, which constitutes the core molecular basis for the heterogeneous inflammatory phenotypes of infectious myocarditis. This chapter elaborates the corresponding TLR recognition patterns and downstream effects according to pathogen categories.

### 2.1 Viral myocarditis: nucleic acid recognition mediated by intracellular TLR3/7/8/9

Viruses activate intracellular TLR receptors relying on their own nucleic acid components, which is the core molecular mechanism for initiating inflammatory cascade reactions in viral myocarditis.

#### 2.1.1 SARS-CoV-2 mediated TLR specific recognition and cross activation

SARS-CoV-2 can invade cardiomyocytes and fibroblasts through the binding of spike protein to host cell ACE2 receptors. The high expression of ACE2 receptors in such cells provides a necessary condition for viral invasion and colonization. In addition to classically activating the TLR3/7/9 pathway, SARS-CoV-2 can also induce a stronger cytokine storm through the ACE2-TLR4 cross-activation pathway, thereby aggravating myocardial inflammatory injury (16, 34). Its spike protein mainly targets and activates TLR7 and NLRP3 inflammasome, and can stably activate innate immune receptors such as TLR3 and MDA-5 at the same time. Moreover, viral RNA and protein can stay in myocardial tissue for a long time (up to 18 months), continuously activating TLR4/9 to mediate chronic inflammatory response (35, 36). Myocardial tissue of fatal COVID-19-infected patients shows an immune phenotype of high TLR4 expression and low TLR9 expression, accompanied by obvious lymphocyte and macrophage infiltration injury (37).

Collectively, the recognition mode of SARS-CoV-2 can be summarized as a complete mechanistic chain: viral spike protein and RNA (PAMPs) → cross-activation of TLR3/7/9 and ACE2-TLR4 → downstream pathways including NF-*κ*B, IRF3 and NLRP3 inflammasome → effector cells including cardiomyocytes, macrophages and endothelial cells → pathological phenotypes of cytokine storm, cardiomyocyte injury and persistent chronic inflammation.

#### 2.1.2 Enterovirus mediated TLR recognition and immune evasion

Enteroviruses are common pathogens inducing viral myocarditis. Coxsackievirus B3 (CVB3) mainly activates the TLR3/4 signaling pathway, in which TLR3 is responsible for recognizing viral double-stranded RNA, and TLR4 is activated by the virus through an indirect pathway (38). Enterovirus A71 (EV-A71), CVB3 and Enterovirus D68 (EV-D68) can evade the antiviral innate immune response mediated by TLR3/7 by cleaving key signaling molecules such as TRIF, TRAF3 and TAK1, so as to complete their own replication and tissue invasion (39, 40). EV-A71 can also widely inhibit the TLR signaling pathway by up-regulating the Sox4 transcription factor, or reduce the ubiquitination level of TBK1 through USP24 to weaken the antiviral effect of the TLR-IRF3 pathway (41, 42). Enterovirus infection can induce autophagy dysfunction in cardiomyocytes, thereby down-regulating TLR7 expression and inhibiting type I interferon production, creating a favorable microenvironment for viral replication (43). CVB3 infection can directly induce the up-regulation of TLR4 expression in cardiomyocytes. TLR4-deficient mice have significantly enhanced resistance to CVB3 infection, and the degree of myocardial inflammation and viral replication level are greatly reduced (44).

The recognition and immune evasion mechanism of enteroviruses follows the chain: viral double-stranded RNA and indirectly activated endogenous ligands (PAMPs/DAMPs) → TLR3/TLR4 → downstream TRIF and MyD88 pathways → cardiomyocytes and immune cells → pathological features of concurrent viral replication, inflammatory injury and immune evasion.

#### 2.1.3 Other viral mediated TLR specific recognition

Influenza virus mainly activates host innate immune responses through TLR7/8, while adenovirus and Epstein–Barr virus take TLR9 as the core recognition receptor to initiate downstream inflammatory signaling pathways. Different viruses have clear specificity in TLR recognition subtypes (1). TLR7 can specifically recognize viral single-stranded RNA, and TLR9 can target viral CpG-DNA, both of which are core receptors for pDCs to mediate antiviral immune responses (45).

In addition to virus-derived single-stranded RNA, extracellular miR-146a-5p can also induce myocardial inflammation and cardiomyocyte dysfunction in mice through TLR7 activation, confirming that TLR7 can respond to endogenous non-viral nucleic acid ligands and participate in the process of sterile myocardial inflammation (46).

After myocardial infarction, TLR7 gene expression is upregulated in ischemic myocardial tissue of both humans and mice. TLR7 knockout mice exhibit reduced incidence of acute cardiac rupture, decreased inflammatory cell infiltration and inflammatory factor expression, attenuated adverse left ventricular remodeling at 28 days after surgery, and improved cardiac function. Bone marrow transplantation experiments further confirm that TLR7 deficiency in bone marrow-derived cells also exerts cardioprotective effects (47).

### 2.2 Bacterial myocarditis: TLR4 and TLR2 mediated specific recognition

#### 2.2.1 Gram-negative bacteria: LPS-TLR4 axis as core pathway

Myocardial injury mediated by gram-negative bacteria takes the LPS-TLR4-MyD88/NF-*κ*B axis as the core signaling pathway, which is the core mediating mechanism of inflammatory injury in septic cardiomyopathy. LPS can directly activate the TLR4-NF-*κ*B signaling pathway in cardiomyocytes, induce the release of pro-inflammatory factors and chemokines such as IL-6, IL-1β and CXCL2, recruit immune cell infiltration and aggravate myocardial injury (11, 48). The intact *Escherichia coli* contains a variety of pathogen-associated molecular patterns, which can simultaneously activate multiple pattern recognition receptors such as TLR4, TLR2 and TLR9. Therefore, single blockade of TLR4 cannot reverse the myocardial inflammatory response induced by *Escherichia coli*, and a multi-target combined intervention strategy is required (48). The LPS-TLR4 axis can also activate heparanase-1 (Hpa-1), cleave endothelial glycocalyx and release damage-associated molecular patterns (DAMPs), further amplifying the inflammatory vicious cycle and forming a cascade reaction of “pathogen activation—TLR—inflammatory amplification” (49). At the same time, this pathway can activate the STING-NLRP3 inflammasome, aggravating myocardial inflammation, apoptosis and contractile dysfunction (50).

In a lipopolysaccharide-induced sepsis model, both protein and mRNA expression of TLR4 are significantly upregulated in the whole heart and left ventricular tissue of mice. MG53 can downregulate TLR4 expression via ubiquitination of ATF2, thereby alleviating sepsis-induced cardiac dysfunction, which further validates the central mediating role of TLR4 in bacterial myocardial injury (51).

The complete mechanistic chain of Gram-negative bacteria-mediated myocardial injury is as follows: lipopolysaccharide (PAMP) → TLR4 → downstream MyD88/NF-*κ*B and STING-NLRP3 pathways → cardiomyocytes, macrophages and endothelial cells → pathological phenotypes of acute inflammatory storm, myocardial systolic dysfunction and endothelial glycocalyx injury.

#### 2.2.2 Gram-positive bacteria: TLR2 mediated core mechanism

Myocardial injury mediated by gram-positive bacteria is mainly realized through the TLR2 pathway. Its cell wall components peptidoglycan and lipoteichoic acid can activate innate immunity through TLR2/1 or TLR2/6 heterodimers, which is directly related to myocardial injury caused by infective endocarditis (18). *Borrelia burgdorferi* can recognize bacterial lipoproteins through TLR2/1, inducing chronic occult myocarditis, which is easy to be missed in clinical practice due to the lack of specific clinical manifestations (52). The concentrations of LPS and (1 → 3)-β-D-glucan in the serum of severe COVID-19 patients are positively correlated with the severity of the disease, suggesting that secondary bacterial infection can further aggravate myocardial injury mediated by SARS-CoV-2 (53).

The mechanistic chain of Gram-positive bacteria-mediated myocardial injury is: peptidoglycan and lipoteichoic acid (PAMPs) → TLR2/1 or TLR2/6 heterodimers → downstream NF-*κ*B pathway → macrophages and cardiomyocytes → pathological phenotypes of chronic occult myocardial injury and myocardial involvement related to infective endocarditis.

### 2.3 Fungal myocarditis: TLR4/Dectin-1 synergistic recognition

Fungal myocarditis is mostly seen in immunosuppressed hosts. Fungi can simultaneously activate TLRs and C-type lectin receptors through cell wall polysaccharide components, thereby producing synergistic pro-inflammatory effects. *Candida β*-glucan and *Aspergillus* mannan activate innate immune responses through TLR2/6 and TLR4 respectively (54). Fungal (1 → 3)-β-D-glucan is activated through the Dectin-1 receptor, forming a synergistic effect with the LPS-TLR4 pathway, which significantly aggravates the myocardial fibrosis process in immunosuppressed hosts (54, 55). *Candida albicans* extract can directly induce cardiovascular inflammatory response through the TLR4 pathway, which is the core pathogenic mechanism of fungal-related myocarditis (30).

The mechanistic chain of fungi-mediated myocardial injury is: cell wall *β*-glucan and mannan (PAMPs) → synergistic recognition by TLR2/6, TLR4 and Dectin-1 → combined activation of downstream inflammatory pathways → macrophages and fibroblasts → pathological phenotype of exacerbated myocardial fibrosis in immunocompromised hosts.

### 2.4 Idiopathic myocarditis: ERV mediated TLR7/9 activation

Overactivation of TLR7 signaling can induce severe hemorrhagic myocarditis in mice. In autoimmune myocarditis models, TLR7 knockout mice are resistant to the onset and progression of myocarditis, suggesting that TLR7 activation is a key driving link in the pathological process of myocarditis and providing direct experimental support for the pathogenic mechanism of ERV-mediated TLR7/9 activation (56).

Abnormal resurrection of endogenous retroviruses (ERVs) is a novel etiology of idiopathic myocarditis. TRIM28 deficiency in cardiomyocytes can directly lead to abnormal activation of ERVs, among which ERV1 is the most significantly activated ERV subtype in failing hearts. ERV resurrection does not activate the type I interferon pathway, but specifically activates the TLR7/9-MyD88-NF-*κ*B inflammator*y* axis, directly inducing fulminant myocarditis. This mechanism provides a new theoretical explanation for idiopathic myocarditis with unknown etiology in the past (57, 58).

The mechanistic chain of ERV-mediated idiopathic myocarditis is: ERV-derived nucleic acid endogenous ligands (DAMP-like) → TLR7/9 → MyD88-NF-*κ*B inflammator*y* axis → cardiomyocytes and infiltrating immune cells → pathological phenotype of fulminant myocarditis.

### 2.5 AAV gene therapy related myocardial injury: TLR mediated innate immune recognition

As a commonly used gene therapy vector in clinical practice, AAV viral vectors can be directly recognized by the host innate immune system, thereby inducing immune-mediated myocardial injury/myocarditis. AAV vector serotype (AAV-5/6/8/9) and high-dose infusion (>1 × 10^12^ vg/kg) are independent risk factors for adverse immune events such as myocarditis. This conclusion further confirms that pathogen/vector-TLR specific recognition is the core rule of myocardial immune injury (15).

It should be clarified that AAV vector-mediated myocardial injury is iatrogenic innate immune activation related to gene therapy, which does not belong to the category of traditional infectious myocarditis. Its TLR recognition mechanism shares common features with innate immune responses induced by viral nucleic acids, which can provide reference for the study of immune regulation in infectious myocarditis.

Collectively, pathogen-TLR recognition patterns present high subtype specificity, leading to distinct downstream signaling cascades and pathological outputs. The detailed recognition spectrum of each pathogen category is summarized in Table 1.

**Table 1:** TLR-specific recognition spectra in infectious myocarditis.

Pathogen type	Core TLR receptors	Key PAMPs/Ligands	Core signaling pathways	Major effector cells	Core pathological & clinical features
RNA/DNA viruses	TLR3/7/8/9, TLR4	Viral dsRNA, ssRNA, CpG-DNA; SARS-CoV-2 spike protein	TLR-NF-*κ*B, TLR-IRF3, NLRP3 inflammasome	Cardiomyocytes, macrophages, pDCs, endothelial cells	Acute inflammatory storm, cytokine release, persistent chronic inflammation (SARS-CoV-2)
Gram-Negative bacteria	TLR4	Lipopolysaccharide (LPS)	TLR4-MyD88-NF-κB, STING-NLRP3 inflammasome	Cardiomyocytes, macrophages, endothelial cells	Septic cardiomyopathy, acute systolic dysfunction, endothelial glycocalyx injury
Gram-Positive bacteria	TLR2/1, TLR2/6	Peptidoglycan, lipoteichoic acid, bacterial lipoproteins	TLR2-NF-κB	Macrophages, cardiomyocytes	Chronic occult myocardial injury, infective endocarditis-related myocardial involvement
Fungi	TLR2/6, TLR4, Dectin-1	Cell wall *β*-glucan, mannan	TLR-Dectin-1 synergistic pathway	Macrophages, cardiac fibroblasts	Exacerbated myocardial fibrosis in immunocompromised hosts
Endogenous retroviruses (ERVs)	TLR7/9	ERV-derived nucleic acids	TLR7/9-MyD88-NF-κB inflammator*y* axis	Cardiomyocytes, infiltrating immune cells	Idiopathic fulminant myocarditis
AAV vectors	Nucleic acid-sensing TLRs	AAV vector nucleic acids	TLR-mediated inflammatory cascade	Cardiac resident and infiltrating immune cells	Onset at 2nd week post-infusion, specificity for muscular/cardiac disease populations

**Figure 1:**
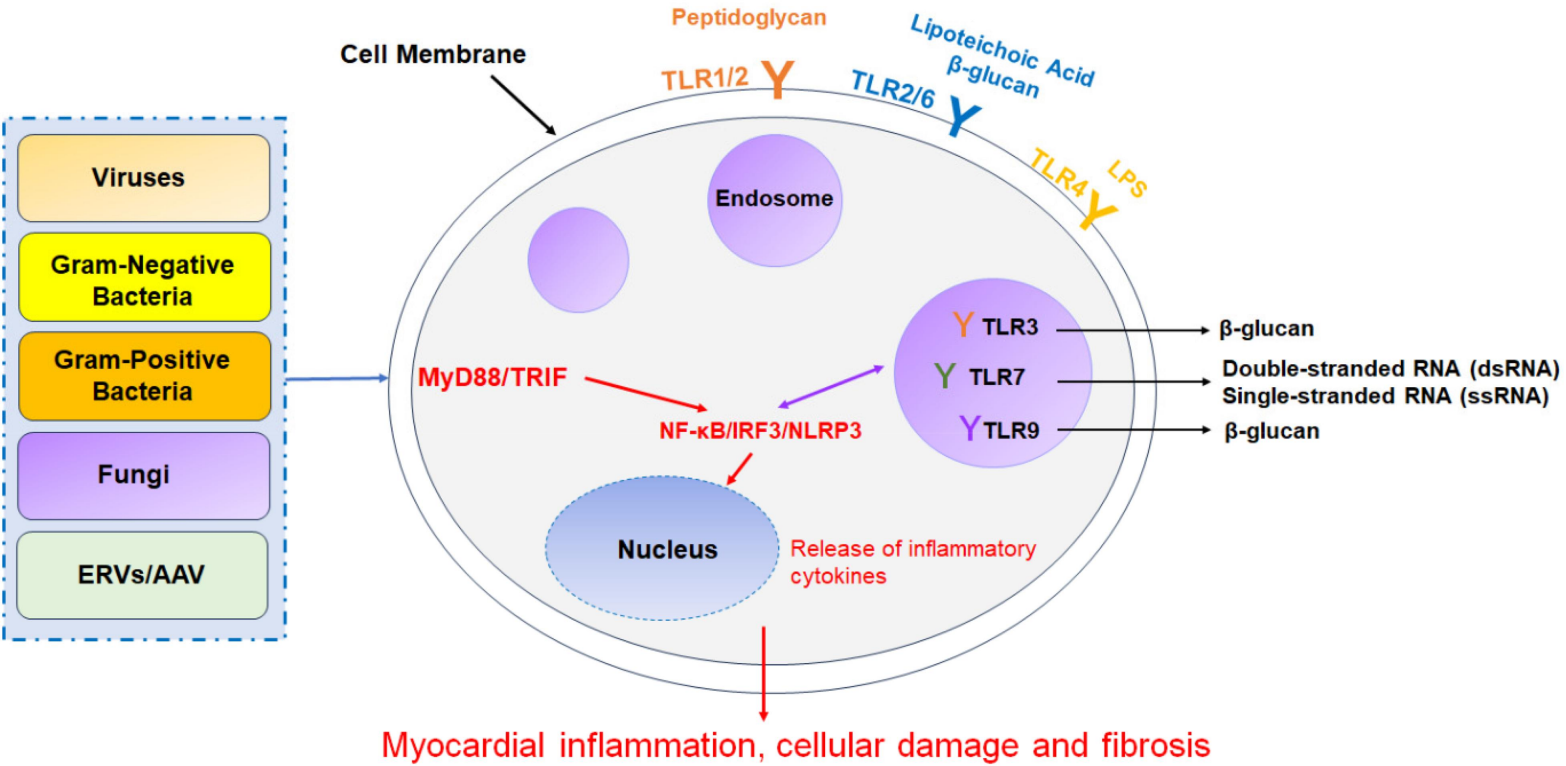
Specific recognition of distinct pathogens by TLRs in cardiomyocytes and subsequent inflammatory activation pathways. This diagram illustrates the specific recognition of pathogen-associated molecular patterns (PAMPs) from five classes of pathogens (viruses, Gram-negative bacteria, Gram-positive bacteria, fungi, ERV/AAV) by distinct TLR subtypes localized on the plasma membrane and endosomes of cardiomyocytes. Membrane-localized TLRs recognize: TLR1/2 heterodimers for peptidoglycan (Gram-positive); TLR2/6 heterodimers for lipoteichoic acid (Gram-positive); TLR4 for LPS (Gram-negative); and TLR2/6 heterodimers for *β*-glucan (Fungi). Endosomal TLRs recognize: TLR3 for viral dsRNA; TLR7/8 for viral ssRNA; and TLR9 for viral CpG-DNA and unmethylated CpG sequences derived from ERVs/AAVs. Ligand recognition triggers MyD88- or TRIF-dependent signaling cascades, leading to the activation of NF-*κ*B, IRF3, and the NLRP3 inflammasome, which ultimately results in the release of inflammatory cytokines, myocardial inflammation, cellular injury, and fibrosis.

In summary, distinct pathogens selectively activate specific TLR subtypes via their unique pathogen-associated molecular patterns (PAMPs), mediating differential initiation and amplification of inflammatory responses. This represents the core molecular basis for the clinical heterogeneity of infectious myocarditis. The complete recognition pattern and downstream signaling pathways are summarized below (Figure 1).

## 3 Spatiotemporal dynamics of TLR signaling in infectious myocarditis

Pathogen-TLR specific recognition determines the intensity and characteristics of inflammatory initiation, while the spatiotemporal dynamic changes of TLR signaling along with disease progression directly regulate the transition from acute inflammation to chronic fibrosis. This chapter analyzes the temporal patterns of TLR signaling and spatial functional transformation according to disease stages.

The activation of TLR signaling does not maintain a constant state, but shows significant spatiotemporal dynamic characteristics with the progression of myocarditis, which is embodied in the sequential changes of rapid initiation in the acute phase, immune deviation in the transitional phase, and continuous pro-fibrosis in the chronic phase. At the same time, it is accompanied by the spatial transformation of TLR functional division between cardiomyocytes and immune cells, and the sequential expression of endogenous negative regulatory networks.

### 3.1 Acute phase (0–7 days): rapid TLR activation and inflammatory storm

The three-stage time classification system adopted in this review (acute phase: 0–7 days, transitional phase: 7–28 days, chronic phase: >28 days) is based on the evolution pattern of viral myocarditis animal models, the natural course characteristics of clinical viral myocarditis, and the scientific statement on the immunopathological process of myocarditis from the European Society of Cardiology (ESC). It is a generally accepted pathological staging framework in the field, used to elaborate the temporal dynamic characteristics of TLR signaling, and does not directly correspond to time nodes for clinical diagnosis and treatment (2, 59). This precise day-scale division of three phases is refined from the ESC consensus pathological staging (2) combined with time-series immune data from viral myocarditis animal models (59), and only serves to illustrate dynamic TLR signaling changes rather than clinical diagnostic criteria.

0–7 days after infection is the acute phase of myocarditis. The expression of TLR3/4/9 is rapidly upregulated to initiate the host innate immune response to clear pathogens, which is a key physiological process of the body's defense response. However, excessive activation of TLRs can induce severe inflammatory storms, causing irreversible myocardial injury, thus showing a clear bidirectional effect of coexistence of physiological defense and pathological injury (37, 60).

There are significant gradient differences in the intensity of acute inflammation induced by different pathogens. The inflammatory response induced by SARS-CoV-2 is much higher than that of ordinary viruses. In the acute phase of sepsis, the LPS-TLR4 axis is rapidly activated, and the phosphorylation level of JNK peaks within 3 h. In the acute phase of viral or ERV infection, the rapid up-regulation of TLR7/9 expression and initiation of innate immune response are the core links of myocardial acute inflammation (57). TLR4/NF-*κ*B signaling is a key upstream mechanism inducing ferroptosis in cardiomyocytes, and ferroptosis can further amplify acute myocardial injury, forming a vicious closed loop of “TLR activation—ferroptosis—aggravated inflammation” (61). The elevated plasma IL-6 level is directly related to the increased infarct size and adverse myocardial remodeling in patients, which can be used as an effective biomarker for TLR-mediated acute myocardial injury (62).

### 3.2 Transitional phase (7–28 days): immune tolerance breakdown and autoimmunity transformation

7–28 days after infection is the transitional phase of myocarditis. At this time, the pathogens have been basically cleared by the body, but damage-associated molecular patterns (HMGB1, mitochondrial DNA, ATP) continuously activate TLR receptors, breaking the myocardial immune tolerance state and completing the key transformation from infectious inflammation to autoimmune inflammation (63, 64). HMGB1 released during ferroptosis can directly activate the TLR4-NF-*κ*B pathway, further amplify the inflammatory response and promote the transformation of the disease course to autoimmunity (61). Mitochondrial DNA can simultaneously activate TLR9 and cGAS-STING pathways, becoming a core threshold molecule driving inflammatory phenotype transformation (65). In post-COVID-19 myocarditis, the long-term retention of the virus in myocardial tissue continuously induces the production of anti-cardiac autoantibodies, accelerating the transformation process from infectious inflammation to autoimmune inflammation (36).

TLR7 deficiency attenuates inflammatory response and adverse ventricular remodeling after myocardial ischemia-reperfusion injury. The underlying mechanism involves driving macrophage polarization toward a reparative (M2-like) phenotype, upregulating efferocytosis-related receptors (CD36, LRP1, MerTK, AXL, Rac1) to enhance apoptotic cell clearance, and thereby promoting inflammation resolution. Meanwhile, TLR7 deficiency can also promote the clearance of neutrophil extracellular traps (NETs) and accelerate the remission of inflammation (66).

### 3.3 Chronic phase (>28 days): sustained TLR activation and fibrotic remodeling

Myocarditis enters the chronic phase more than 28 days after infection. Sustained activation of TLR-MyD88-NF-*κ*B signaling drives the activation of myocardial fibroblasts, promotes the secretion of TGF-*β*1 and collagen deposition, and finally induces myocardial fibrosis and adverse myocardial remodeling (67, 68). In the chronic phase of post-COVID-19 myocarditis, TLR4/9 is continuously highly expressed, and CD68 + macrophages continuously infiltrate myocardial tissue, further aggravating the degree of myocardial fibrosis (36). Fungal *β*-glucan and Dectin-1 synergistic action can induce chronic myocardial fibrosis (54). Ferroptosis forms a positive feedback cycle with the TLR4/NF-*κ*B pathway, participating in the chronic myocardial remodeling process (69).

### 3.4 Intercellular communication: spatiotemporal functional division of TLRs

In the acute phase, TLRs in cardiomyocytes first sense pathogens or damage signals, release pro-inflammatory factors and chemokines, and recruit immune cells such as macrophages and neutrophils to infiltrate myocardial tissue (59). In the chronic phase, TLR receptors in macrophages and fibroblasts become the core source of inflammatory signals, continuously amplifying inflammatory signals and driving the fibrosis process (59). Exosomes released by cardiomyocytes can carry viral nucleic acids and damage-associated molecular patterns, remotely activate TLR signals in adjacent cells, and expand the scope of inflammatory injury (70).

Clusterin⁺ cardiomyocytes induced by myocardial injury can secrete Clusterin protein, which reprograms macrophage function by binding to TLR4 on the macrophage surface, exerting effects of inhibiting inflammation and promoting cardiac regeneration. This confirms that cardiomyocytes and immune cells can achieve intercellular communication and functional regulation through TLR ligand-receptor interaction (71).

In a model of angiotensin Ⅱ-induced sterile myocardial injury, small extracellular vesicles derived from cardiomyocytes are enriched with let-7b-5p, which can paracrinally activate TLR7/MyD88/NF-*κ*B signaling in resident cardiac macrophages and fibroblasts, promote inflammatory factor expression, fibroblast transdifferentiation and adverse ventricular remodeling. Knockdown of let-7b-5p, TLR7 or MyD88 can all abolish this effect (72).

### 3.5 Stage-specific negative regulation of TLR signaling

In the acute phase, A20, SOCS1, SIGIRR and miR-146a are rapidly up-regulated to limit the excessive amplification of inflammatory storms, preventing excessive inflammatory response from causing irreversible myocardial injury (30, 38, 73). These endogenous negative regulatory molecules constitute the first line of defense to maintain cardiac immune homeostasis. In the chronic phase, the negative regulatory network initiated in the acute phase is inactivated, leading to the persistence of inflammatory signals and continuous progression of myocardial fibrosis (73). Taken together, TLR signaling undergoes sequential dynamic evolution with disease progression, accompanied by coordinated shifts in both temporal activation patterns and spatial cellular functions. The core features across different disease stages are outlined in Table 2.

**Table 2:** Spatiotemporal dynamic characteristics of TLR signaling.

Disease stage	Time window	Core TLR alterations	Dominant driving factors	Spatial functional shift	Core pathological outcome
Acute phase	0–7 days	Rapid upregulation of TLR3/4/7/9	Pathogen PAMPs, initial DAMPs release	Cardiomyocytes act as primary sensing cells and recruit infiltrating immune cells	Inflammatory storm, acute myocardial injury
Transitional phase	7–28 days	Sustained TLR activation mediated by DAMPs	HMGB1, mitochondrial DNA, persistent viral antigens	Dominance shifts to innate/adaptive immune cells; cardiac immune tolerance breaks down	Immune deviation, transition to autoimmunity
Chronic phase	>28 days	Sustained high expression of TLR2/4/9	Persistent DAMPs, pro-fibrotic signaling	Macrophages and fibroblasts become the main source of inflammatory signals	Myocardial fibrosis, adverse remodeling, heart failure

**Figure 2:**
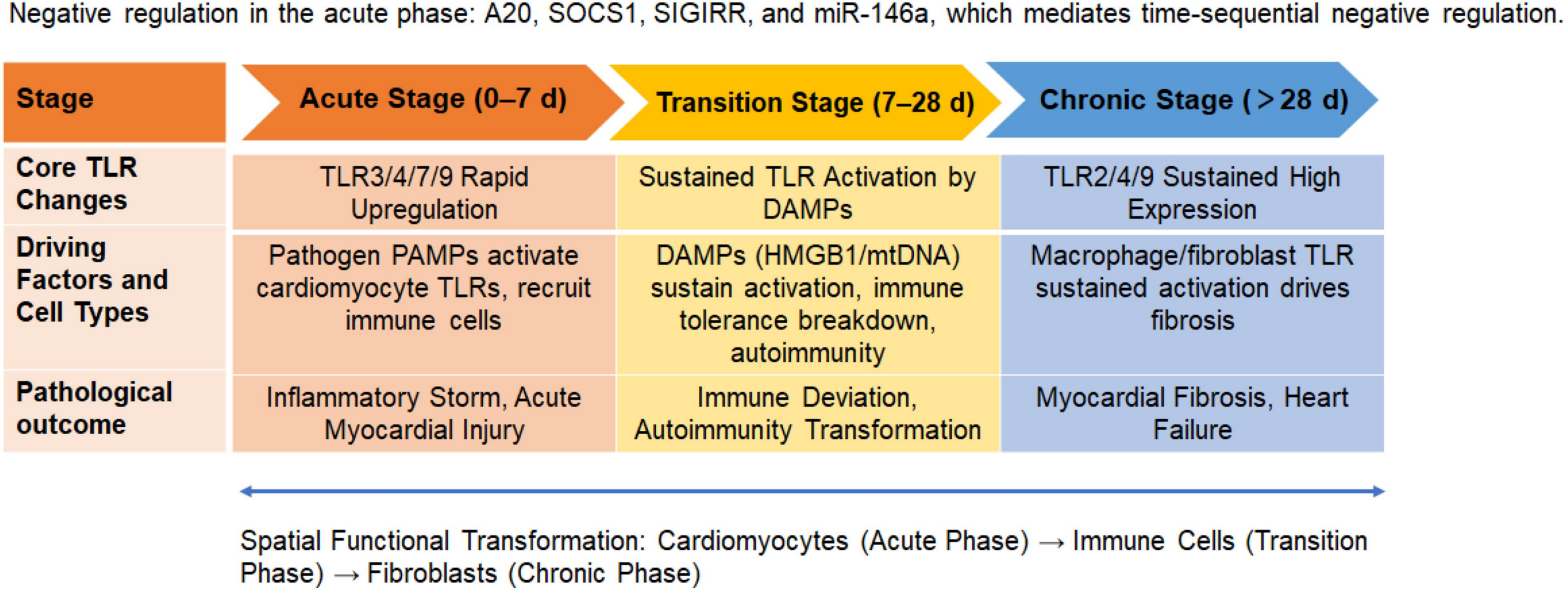
Spatiotemporal dynamic regulation of TLR signaling and pathological outcomes. This figure depicts the spatiotemporal dynamics of TLR signaling throughout the three phases of infectious myocarditis, illustrating changes in TLR profiles, driving factors, cellular functional transitions, and pathological outcomes. **Acute Phase (0–7 d):** Pathogen PAMPs activate TLR3/4/7/9 on cardiomyocytes, triggering an inflammatory storm and acute myocardial injury; this phase is counterbalanced by negative regulators such as A20, SOCS1, SIGIRR, and miR-146a. **Transitional Phase (7–28 d):** Damage-associated molecular patterns (DAMPs, e.g., HMGB1, mitochondrial DNA) sustain TLR activation, breaking immune tolerance and driving immune deviation and autoimmunity transformation. **Chronic Phase (>28 d):** Sustained high expression of TLR2/4/9 in macrophages and fibroblasts drives myocardial fibrosis and heart failure. Spatially, the functional relay transitions from cardiomyocytes (acute) to immune cells (transitional) and finally to fibroblasts (chronic).

Overall, the activation intensity, cellular sources, and functional effects of TLR signaling exhibit significant temporal evolution as the disease progresses, accompanied by spatial functional relay between cell types, collectively driving the transition from acute inflammation to chronic fibrosis. The complete dynamic regulatory patterns are outlined below (Figure 2).

## 4 TLR-targeted intervention strategies and clinical translation

Based on the pathogen recognition specificity and spatiotemporal regulatory characteristics of TLRs, TLR-targeted intervention for infectious myocarditis has evolved from broad-spectrum non-specific blockade to refined strategies oriented by pathogen type and precise stage-specific regulation. This chapter summarizes available intervention approaches, clinical translation value and existing bottlenecks.

### 4.1 Core principles of TLR-targeted intervention

TLR intervention should follow pathogen specificity: viral/ERV-related myocarditis preferentially targets and blocks TLR7/9, bacterial/septic cardiomyopathy preferentially blocks TLR4, and gram-positive bacteria-related myocardial injury takes the TLR2 pathway as the core intervention target (52, 57, 74). COVID-19 myocarditis needs to simultaneously target the ACE2-TLR4 cross-activation pathway, and fungal myocarditis needs to combinedly block TLR2/6/TLR4 and Dectin-1 (54, 74).

Intervention should match disease stage to avoid invalid or reverse injury caused by non-sequential intervention (75).

### 4.2 TLR-targeted intervention methods

#### 4.2.1 TLR subtype-specific small molecule inhibitors

TLR4 small molecule inhibitors TAK-242 and CLI-095 can effectively alleviate LPS-induced myocardial inflammation and cardiac function injury, and CLI-095 can also reduce the myocardial infarct size caused by ischemia-reperfusion (48, 76). TAK-242 can reverse PCSK9-induced myocardial fibrosis and inflammatory activation (77). TLR7/9 inhibitor NSC4375 can specifically block the ERV-TLR7/9 activation axis and improve myocardial injury (57). Most TLR inhibitors have clear temporal effect limitations and are only effective in early intervention (75, 77).

In addition to experimental small-molecule inhibitors, clinically marketed hydroxychloroquine (HCQ) and chloroquine (CQ) are classic endosomal TLR modulators. As 4-aminoquinoline antimalarial and immunomodulatory agents, they accumulate in acidic endosomes/lysosomes to raise the luminal pH, and selectively inhibit nucleic acid ligand activation of endosomal TLR9 (recognizing CpG-DNA) and TLR7/TLR8 (recognizing single-stranded RNA) at low concentrations (≤20 *μ*M), blocking downstream MyD88-dependent type Ⅰ interferon and inflammatory cytokine production. They are widely used TLR7/TLR9 antagonists in autoimmune diseases such as systemic lupus erythematosus and rheumatoid arthritis (78, 79).

Basic studies have shown that hydroxychloroquine inhibits macrophage activation and MAPK phosphorylation by suppressing the endosomal TLR9 signaling pathway, and attenuates renal tubulointerstitial fibrosis after ischemia-reperfusion injury in mice. The anti-inflammatory and anti-fibrotic effects of hydroxychloroquine are abolished in TLR9 knockout mice, confirming that its organ protective effect depends on TLR9 inhibition, which provides mechanistic reference for its application in myocardial ischemia and inflammatory injury (80).

In a mouse model of myocardial ischemia-reperfusion injury, hydroxychloroquine, as a TLR7/TLR9 antagonist, attenuates myocardial injury by inhibiting the endosomal TLR9-type Ⅰ interferon pathway. Both pretreatment and administration before reperfusion can reduce infarct size and lower IFN-α/IFN-*β* levels in plasma and myocardial perfusate. TLR9 knockout mice present the same phenotype and hydroxychloroquine no longer produces additional benefits, clarifying its core mechanism of cardioprotection via TLR9 inhibition (81).

#### 4.2.2 Downstream pathway targeted intervention

Inhibiting the phosphorylation level of JNK downstream of TLR4 can effectively reverse myocardial inflammation and cardiac dysfunction in septic cardiomyopathy (76). Activating AKT2 can repair the innate immune defect of CVB3 myocarditis, inhibit the compensatory up-regulation of TLR4, reduce viral titer and alleviate myocardial injury (44). Ferroptosis inhibitors can down-regulate the TLR4/NF-*κ*B pathway and alleviate myocardial function injury (69). Endothelial cell exosomes can targetedly inhibit the TLR-NF-*κ*B axis, reducing myocardial inflammation (64). Platelet-derived extracellular vesicles also exert therapeutic effects in viral myocarditis by modulating innate immune signaling and reducing myocardial inflammatory injury (82).

### 4.3 Special etiology combined intervention

AAV9-TRIM28 gene therapy can inhibit abnormal ERV resurrection from the source, thereby blocking the pathological activation of TLR7/9 (57). Perioperative immunosuppressive regimens can reduce the risk of AAV-related myocarditis (15). Omega-3 polyunsaturated fatty acids can down-regulate TLR2/9 expression and improve myocardial inflammation in patients with heart failure (83).

### 4.4 Biomarkers for diagnosis and efficacy prediction

TLR4 can be used as a biomarker for predicting the efficacy of immunosuppressive therapy in post-COVID-19 myocarditis (36, 84). sTLR2/4, Presepsin and ERV RNA can be used as specific biomarkers for prognosis evaluation and efficacy monitoring (85). Presepsin can be used as an independent predictive biomarker for acute myocarditis (86). Gender affects TLR4 activity and myocarditis incidence, which should be included in the evaluation system (87, 88).

### 4.5 Clinical translation bottlenecks

The core bottlenecks include unclear precise therapeutic window, lack of standardized patient stratification system, and insufficient specific biomarkers for early diagnosis and efficacy monitoring (84, 85, 89). These problems seriously restrict the clinical transformation and application of TLR-targeted therapy. Overall, TLR-targeted interventions cover multiple levels from receptor blockade to downstream pathway modulation, while facing common obstacles in clinical translation. The specific strategies and corresponding bottlenecks are detailed in Table 3.

**Table 3:** TLR-targeted intervention strategies and clinical translation bottlenecks.

Intervention Type	Representative Agents	Target TLRs/Pathways	Drug Category	Core Efficacy	Clinical Translation Bottlenecks
TLR subtype-specific inhibitors	TAK-242, CLI-095, NSC4375, Hydroxychloroquine	TLR4, TLR7/9	Experimental (TAK-242, CLI-095, NSC4375); Clinically available (Hydroxychloroquine)	Attenuate inflammatory response, inhibit myocardial fibrosis	Narrow therapeutic window; lack of myocarditis-specific clinical trials
Downstream pathway intervention	JNK inhibitors, AKT2 activators, ferroptosis inhibitors, endothelial exosomes	TLR4 downstream cascades, ferroptosis-TLR4 loop	Experimental	Reverse myocardial dysfunction, promote inflammation resolution	Off-target effects; unclear optimal intervention timing
Etiology-specific combined intervention	AAV9-TRIM28 gene therapy, perioperative immunosuppression, Omega-3 PUFAs	TLR7/9 (ERV axis), TLR2/9	Gene therapy/adjunctive therapy	Inhibit ERV activation; reduce AAV-related myocarditis risk	Safety concerns of gene therapy; insufficient evidence for myocarditis indication
Biomarker-guided therapy	sTLR2/4, Presepsin, ERV RNA	—	Diagnostic/monitoring biomarkers	Prognosis evaluation, efficacy monitoring	Not included in routine clinical detection

**Figure 3:**
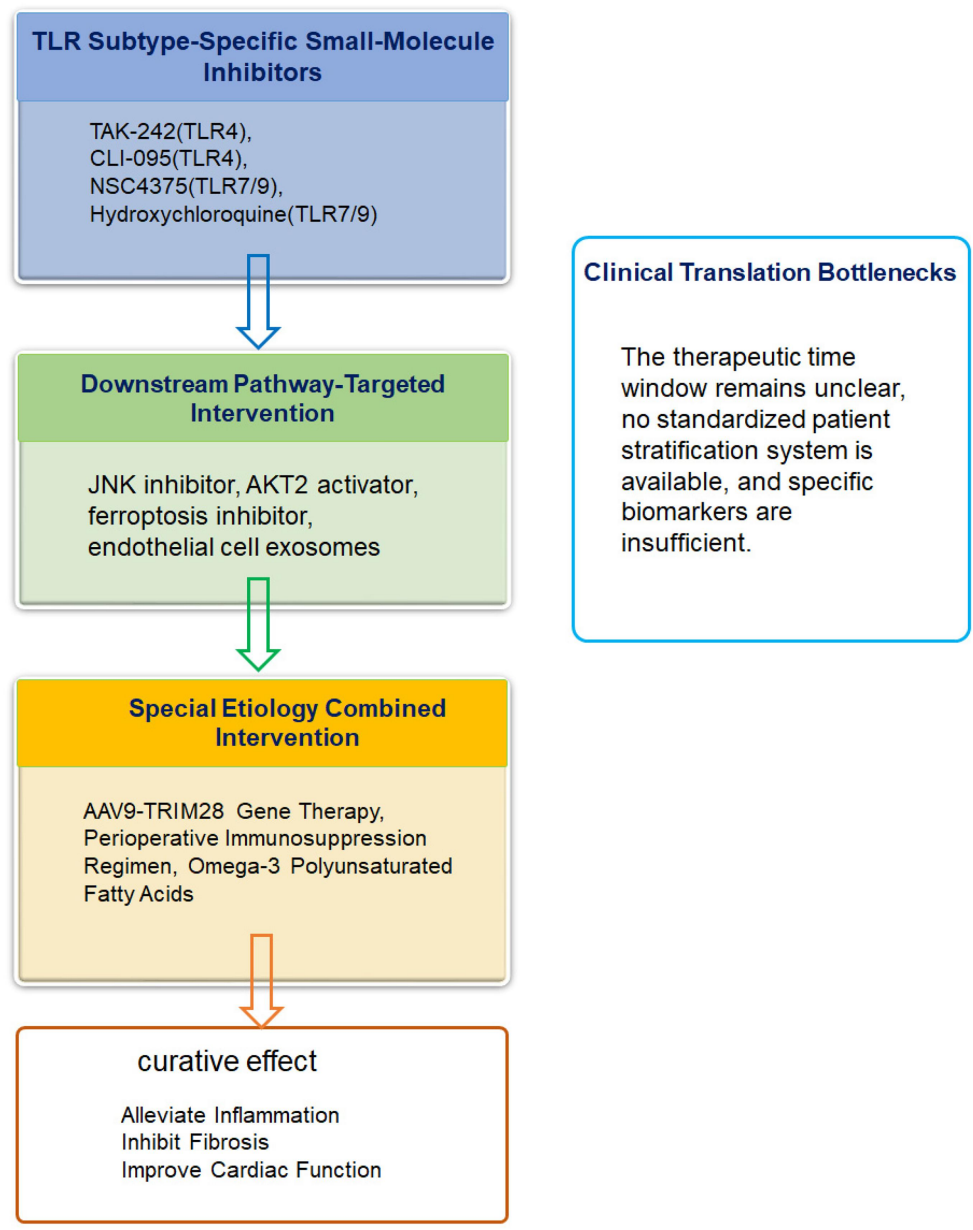
TLR-targeted intervention strategies and core therapeutic effects in infectious myocarditis. This diagram illustrates the hierarchical TLR-targeted intervention strategies from upstream receptors to downstream pathways and their clinical translation status in infectious myocarditis. **Left-side intervention hierarchy:** (1) **Upstream targets:** TLR subtype-specific small-molecule inhibitors (e.g., TAK-242/CLI-095 for TLR4; NSC4375/Hydroxychloroquine for TLR7/9); (2) **Midstream targets:** Downstream pathway-targeted interventions (e.g., JNK inhibitors, AKT2 activators, ferroptosis inhibitors, endothelial cell exosomes); (3) **Etiological layer:** Special etiology combined interventions (e.g., AAV9-TRIM28 gene therapy, perioperative immunosuppression regimens, Omega-3 polyunsaturated fatty acids). **Therapeutic output:** These three layers converge to achieve three core therapeutic effects: alleviating inflammation, inhibiting fibrosis, and improving cardiac function. **Right-side bottlenecks:** Current clinical translation faces three major challenges: unclear therapeutic time windows, lack of a standardized patient stratification system, and insufficient specific biomarkers.

In conclusion, TLR-targeted interventions have evolved into a multilayered strategic system spanning from receptor subtypes to downstream pathways and from single blockade to etiology-combined approaches. However, multiple practical bottlenecks remain for clinical implementation. The overall intervention framework and translational status are summarized below (Figure 3).

## 5 Conclusions and perspectives

### 5.1 Conclusions

Pathogen-TLR matching serves as the fundamental determinant of inflammatory phenotype, reaction intensity and clinical prognosis in infectious myocarditis. Distinct pathogens activate specific TLR subtypes via unique PAMPs, mediate divergent initiation and amplification of inflammatory responses, and ultimately lead to prominent clinical heterogeneity of myocarditis (2). The spatiotemporal dynamics of TLR signaling act as the central mechanism governing disease progression. In the acute phase, TLR activation rapidly triggers anti-infective defensive responses; during the transitional stage, sustained DAMP-induced TLR activation breaks cardiac immune tolerance; in the chronic phase, excessive TLR signaling drives the progression of myocardial fibrosis. In this context, TLR-targeted intervention should strictly follow the core principles of pathogen orientation and staged precise regulation (59). Furthermore, the ERV-TLR7/9 activation axis uncovers a novel molecular mechanism underlying idiopathic myocarditis, while AAV- and ICI-associated myocarditis further broaden the disease spectrum mediated by TLR signaling (15, 57).

### 5.2 Perspectives

From an extended perspective, ischemic myocardial injury and infectious myocarditis share the core mechanism of TLR-mediated innate immune inflammation. Ischemia-reperfusion or permanent coronary ligation leads to massive death of cardiomyocytes, releasing damage-associated molecular patterns including HMGB1, HSP60, S100A8/A9, oxidized low-density lipoprotein, extracellular RNA and mitochondrial DNA. These molecules can activate TLR4 and TLR2 on the surface of resident and infiltrating immune cells, initiate NF-*κ*B activation, pro-inflammatory cytokine production and NLRP3 inflammasome activation via the MyD88-dependent pathway, which constitutes the core innate immune mechanism of early inflammation amplification and adverse left ventricular remodeling after myocardial infarction. TLR4 deficiency or signaling inhibition can reduce infarct size and attenuate adverse remodeling in mice after myocardial infarction, while TLR2 deficiency or blockade can alleviate collagen deposition and fibrosis (90).

Endosomal TLRs also participate in injury amplification after myocardial infarction: single-stranded RNA released from dead cardiomyocytes can be recognized by endosomal TLR7 in leukocytes and activate pro-inflammatory signals, and TLR7 gene knockout can reduce cardiac rupture and adverse remodeling after myocardial infarction. Released mitochondrial DNA can act as an endogenous ligand to activate endosomal TLR9, and both TLR9 gene knockout and hydroxychloroquine (an endosomal inhibitor of TLR7/TLR9) can attenuate myocardial ischemia-reperfusion injury (91).

HMGB1 released from necrotic cardiomyocytes after myocardial infarction triggers early inflammation by activating TLR4 signaling in macrophages. USP9X can inhibit excessive TLR signaling activation by stabilizing TRAFD1 (a negative feedback regulator of TLR-NF-*κ*B), suggesting that ischemic myocardial infarction and infectious myocarditis share the TLR-MyD88-NF-*κ*B core axis and negative feedback regulatory nodes (92). Targeting TLR4 with RP105 has also been shown to attenuate myocardial ischemia-reperfusion injury via inhibiting the TLR4/TRIF pathway, further supporting the translational potential of TLR-targeted strategies in myocardial injury of different etiologies (93).

Future research priorities lie in exploring the cross-regulatory mechanisms between ERV-TLR and mitochondrial DNA-TLR axes, screening TLR-specific biomarkers to establish non-invasive evaluation systems, and developing TLR subtype- and stage-specific small-molecule inhibitors. Such efforts aim to advance the transformation of infectious myocarditis therapy from empirical anti-inflammatory strategies to precise immune modulation, and provide solid theoretical support and practical insights for clinical diagnostic and therapeutic innovation.

## References

[1] Favere K Bosman M Klingel K Heymans S Van Linthout S Delputte PL. Toll-like receptors: are they taking a toll on the heart in viral myocarditis? Viruses. (2021) 13(6):1003. 10.3390/v13061003 34072044 PMC8227433

[2] Tschöpe C Ammirati E Bozkurt B Caforio ALP Cooper LT Felix SB. Myocarditis and inflammatory cardiomyopathy: current evidence and future directions. Nat Rev Cardiol. (2021) 18(3):169–93. 10.1038/s41569-020-00435-x 33046850 PMC7548534

[3] Boehmer TK Kompaniyets L Lavery AM Hsu J Ko JY Yusuf H. Association between COVID-19 and myocarditis using hospital-based administrative data—United States, March 2020–January 2021. Morb Mortal Wkly Rep. (2021) 70(35):1228–32. 10.15585/mmwr.mm7035e5 PMC8422872 34473684

[4] Gyöngyösi M Alcaide P Asselbergs FW Brundel BJJM Camici GG Martins PDC. Long COVID and the cardiovascular system–elucidating causes and cellular mechanisms. Cardiovasc Res. (2023) 119(2):336–56. 10.1093/cvr/cvac115 35875883 PMC9384470

[5] Isath A Malik A Bandyopadhyay D Goel A Hajra A Contreras J. A comparison of COVID-19 and influenza-associated myocarditis: a nationwide study in the United States. Curr Probl Cardiol. (2023) 48(1):101680. 10.1016/j.cpcardiol.2023.101680 36918088 PMC10008141

[6] Nicacio JM Gomes OV Carmo RF Nunes SLP Rocha JRCF Souza CDFD. Heart disease and arboviruses: a systematic review and meta-analysis. Viruses. (2022) 14(9):1988. 10.3390/v14091988 36146794 PMC9502577

[7] Giugni FR Aiello VD Faria CS Pour SZ Cunha MDP Giugni MV. Understanding yellow fever-associated myocardial injury: an autopsy study. EBioMedicine. (2023) 96:104810. 10.1016/j.ebiom.2023.104810 37757571 PMC10550587

[8] Huang S Zhang C Li J Dai Z Huang J Deng F. Designing a multi-epitope vaccine against coxsackievirus B based on immunoinformatics approaches. Front Immunol. (2022) 13:933594. 10.3389/fimmu.2022.933594 36439191 PMC9682020

[9] Simões MP Hodcroft EB Simmonds P Albert J Alidjinou EK Ambert-Balay K. Epidemiological and clinical insights into the enterovirus D68 upsurge in Europe 2021–2022. J Infect Dis. (2024) 230(6):e917–28. 10.1093/infdis/jiae154 38547499 PMC11481312

[10] Caforio AL Pankuweit S Arbustini E Basso C Gimeno-Blanes J Felix SB. Current state of knowledge on aetiology, diagnosis, management, and therapy of myocarditis: a position statement of the European Society of Cardiology. Eur Heart J. (2013) 34(33):2636–48. 10.1093/eurheartj/eht210 23824828

[11] Zhou ZK Yu MS Shou ST Chai Y-F Liu Y-C. Interaction between gut-heart axis in sepsis-induced cardiomyopathy. Pharmacol Res. (2025) 217:107806. 10.1016/j.phrs.2025.107806 40449812

[12] Rudd KE Johnson SC Agesa KM Shackelford KA Tsoi D Kievlan DR. Global, regional, and national sepsis incidence and mortality, 1990–2017: a systematic analysis for the global burden of disease study 2017. Lancet. (2020) 395(10225):200–11. 10.1016/S0140-6736(19)32989-7 31954465 PMC6970225

[13] Umemura Y Ogura H Takuma K Fujishima S Abe T Kushimoto S. Current spectrum of causative pathogens in sepsis: a prospective nationwide cohort study in Japan. Int J Infect Dis. (2021) 103:343–51. 10.1016/j.ijid.2020.11.168 33221519

[14] Patel RP Parikh R Gunturu KS Tariq RZ Dani SS Ganatra S. Cardiotoxicity of immune checkpoint inhibitors. Curr Oncol Rep. (2021) 23(7):79. 10.1007/s11912-021-01070-6 33937956 PMC8088903

[15] Maurizi N Ammirati E Silver E Hong K Bui Q Argirò A. Incidence, timing, and clinical significance of adverse immune events after gene replacement therapy: a systematic review and meta-analysis. Mol Ther. (2026) 34(3):1340–51. 10.1016/j.ymthe.2026.01.004 41520173 PMC12974193

[16] Boulos PK Freeman SV Henry TD Mahmud E Messenger JC. Interaction of COVID-19 with common cardiovascular disorders. Circ Res. (2023) 132(10):1259–71. 10.1161/CIRCRESAHA.122.321952 37167359 PMC10171313

[17] Kosyakovsky LB Angriman F Katz E Adhikari NK Godoy LC Marshall JC. Association between sepsis survivorship and long-term cardiovascular outcomes in adults: a systematic review and meta-analysis. Intensive Care Med. (2021) 47(9):931–42. 10.1007/s00134-021-06479-y 34373953

[18] Bezhaeva T Karper J Quax PHA de Vries M. The intriguing role of TLR accessory molecules in cardiovascular health and disease. Front Cardiovasc Med. (2022) 9:820962. 10.3389/fcvm.2022.820962 35237675 PMC8884272

[19] Takeda K Akira S. TLR signaling pathways. Semin Immunopathol. (2004) 16(1):3–9. 10.1016/j.smim.2003.10.003 14751757

[20] Prabhu SD Frangogiannis NG. The biological basis for cardiac repair after myocardial infarction: from inflammation to fibrosis. Circ Res. (2016) 119(1):91–112. 10.1161/CIRCRESAHA.116.303577 27340270 PMC4922528

[21] Vaez H Soraya H Garjani A Gholikhani T. Toll-like receptor 4 (TLR4) and AMPK relevance in cardiovascular disease. Adv Pharm Bull. (2021) 13(1):36–47. 10.34172/apb.2023.004 36721803 PMC9871286

[22] Feng Y Chao W. Toll-like receptors and myocardial inflammation. Int J Inflamm. (2011) 2011:170352. 10.4061/2011/170352 PMCID:PMC3182762 PMC3182762 21977329

[23] Samad T Wu SM. Single cell RNA sequencing approaches to cardiac development and congenital heart disease. Semin Cell Dev Biol. (2021) 118:129–35. 10.1016/j.semcdb.2021.04.023 34006454 PMC8434959

[24] Jin K-J Pan L Huang C-X Yin C Wang Y Zhang J. Inflammation-related factors S100A9 and TLR2 in cardiomyocyte hypertrophy. Curr Med Sci. (2025) 45(4):819–30. 10.1007/s11596-025-00096-2 40694254

[25] Zhao R Xie Y Xu K Liu Y Chen R Zhao S. 10-hydroxydec-2-enoic Acid alleviates post-myocardial infarction inflammation and oxidative stress by modulating the TLR4 signaling axis. Phytomedicine. (2025) 148:157457. 10.1016/j.phymed.2025.157457 41175588

[26] Dai C Sun J Yang G Zhang C Zhang Y Song Q. The total xanthones from Gentianella acuta alleviate acute myocardial infarction by targeting BRD4-mediated cardiomyocyte pyroptosis and inflammation. Phytomedicine. (2025) 147:157156. 10.1016/j.phymed.2025.157156 40845590

[27] Sreejit G Abdel-Latif A Athmanathan B Annabathula R Dhyani A Noothi SK. Neutrophil-derived S100A8/A9 amplify granulopoiesis after myocardial infarction. Circulation. (2020) 141(13):1080–94. 10.1161/CIRCULATIONAHA.119.043833 31941367 PMC7122461

[28] Mann-Nüttel R Ali S Petzsch P Köhrer K Alferink J Scheu S. The transcription factor reservoir and chromatin landscape in activated plasmacytoid dendritic cells. BMC Genom Data. (2021) 22(1):37. 10.1186/s12863-021-00991-2 34544361 PMC8454182

[29] Zheng X Chen H. Roles of autophagy in sepsis-induced myocardial dysfunction: a comprehensive review. Am J Transl Res. (2026) 18(1):64–76. 10.62347/SZYG2334 41676277 PMC12886153

[30] Wen Z Xia Y Zhang Y He Y Niu C Wu R. SIGIRR-caspase-8 signaling mediates endothelial apoptosis in Kawasaki disease. Ital J Pediatr. (2023) 49(1):2. 10.1186/s13052-022-01401-8 36600293 PMC9811794

[31] O'Neill LA Sheedy FJ McCoy CE. MicroRNAs: the fine-tuners of toll-like receptor signaling. Nat Rev Immunol. (2011) 11(3):163–73. 10.1038/nri2957 21331081

[32] Yang Y Lv J Jiang S Ma Z Wang D Hu W. The emerging role of toll-like receptor 4 in myocardial inflammation. Cell Death Dis. (2016) 7(3):e2234. 10.1038/cddis.2016.140 27228349 PMC4917669

[33] Zheng SY Dong JZ. Role of toll-like receptors and Th responses in viral myocarditis. Front Immunol. (2022) 13:843891. 10.3389/fimmu.2022.843891 35514979 PMC9062100

[34] Gheblawi M Wang K Viveiros A Nguyen Q Zhong J-C Turner AJ. Angiotensin-converting enzyme 2: SARS-CoV-2 receptor and regulator of the renin-angiotensin system: implications for understanding coronavirus diseases 2019 (COVID-19) morbidity. Circ Res. (2020) 126(11):1456–74. 10.1161/CIRCRESAHA.120.317015 32264791 PMC7188049

[35] Bortolotti D Gentili V Rizzo S Schiuma G Beltrami S Strazzabosco G. TLR3 And TLR7 RNA sensor activation during SARS-CoV-2 infection. Microorganisms. (2021) 9(9):1820. 10.3390/microorganisms9091820 34576716 PMC8465566

[36] Blagova OV Kogan EA Novosadov VM Bryukhanov VA Zharkov NV. Post-COVID versus non-COVID myocarditis: comparison of morphological activity, toll-like receptor distribution and responses to immunosuppressive therapy. Front Biosci. (2025) 17(2):28262. 10.31083/FBS28262 40613204

[37] Kogan EA Berezovskiy YS Blagova OV Kukleva A Semyonova L Gretsov E. Morphologically, immunohistochemically and PCR proven lymphocytic viral peri-, endo-, myocarditis in patients with fatal COVID-19. Diagn Pathol. (2022) 17(1):31. 10.1186/s13000-022-01207-6 35177093 PMC8851780

[38] Fei Y Chaulagain A Wang T Chen Y Liu J Yi M. MiR-146a down-regulates inflammatory response by targeting TLR3 and TRAF6 in coxsackievirus B infection. RNA. (2020) 26(1):91–100. 10.1261/rna.071985.119 31676570 PMC6913124

[39] Yin Y Chi X Feng Y Jiang Q. The strategies and mechanisms of enteroviruses to evade innate immunity. Front Cell Infect Microbiol. (2025) 15:1636104. 10.3389/fcimb.2025.1636104 40822588 PMC12350268

[40] Kang J Pang Z Zhou Z Li X Liu S Cheng J. Enterovirus D68 protease 2A(pro) targets TRAF3 to subvert host innate immune responses. J Virol. (2021) 95(3):e01856–20. 10.1128/JVI.01856-20 33148796 PMC7925118

[41] Shang J Zheng Y Mo J Wang W Luo Z Li Y. Sox4 represses host innate immunity to facilitate pathogen infection via hijacking the TLR signaling networks. Virulence. (2021) 12(1):704–22. 10.1080/21505594.2021.1882775 33517839 PMC7894441

[42] Zang L Gu J Yang X Yuan Y Guo H Zhou W. Ubiquitin specific protease 24 promotes EV71 infection by restricting K63-linked polyubiquitination of TBK1. Virol Sin. (2023) 38(1):75–83. 10.1016/j.virs.2022.11.001 36334706 PMC10006192

[43] Wei J Lv J Wang T Gu W Luo Y Feng H. Recent progress in innate immune responses to enterovirus A71 and viral evasion strategies. Int J Mol Sci. (2024) 25(15):5688. 10.3390/ijms25115688 38891876 PMC11172324

[44] Kim SH Shin HH Kim JH Park J-H Jeon E-S Lim B-K. Protein kinase B2 (PKB2/AKT2) is essential for host protection in CVB3-induced acute viral myocarditis. Int J Mol Sci. (2022) 23(3):1489. 10.3390/ijms23031489 35163412 PMC8836114

[45] Hornung V Rothenfusser S Britsch S Krug A Jahrsdörfer B Giese T. Quantitative expression of toll-like receptor 1–10 mRNA in cellular subsets of human peripheral blood mononuclear cells. J Immunol. (2002) 168(9):4531–7. 10.4049/jimmunol.168.9.4531 11970999

[46] Shimada BK Yang Y Zhu J Wang S Suen A Kronstadt SM. Extracellular miR-146a-5p induces cardiac innate immune response and cardiomyocyte dysfunction. Immunohorizons. (2020) 4(9):561–72. 10.4049/immunohorizons.2000075 32958516 PMC7754174

[47] de Kleijn DPV Chong SY Wang X Yatim SMJM Fairhurst A-M Vernooij F. Toll-like receptor 7 deficiency promotes survival and reduces adverse left ventricular remodelling after myocardial infarction. Cardiovasc Res. (2019) 115(12):1791–803. 10.1093/cvr/cvz057 30830156

[48] Wiger CW Ranheim T Arnesen H Vaage J Pischke SE Yndestad A. TLR4 inhibition attenuates LPS-induced proinflammatory signaling and cytokine release in mouse hearts and cardiomyocytes. Immunity Inflamm Dis. (2025) 13:e70133. 10.1002/iid3.70133 PMC11760985 39853914

[49] Pape T Hunkemöller AM Kumpers P Haller H David S Stahl K. Targeting the “sweet spot” in septic shock: a perspective on the endothelial glycocalyx regulating proteins heparanase-1 and -2. Matrix Biol Plus. (2021) 12:100095. 10.1016/j.mbplus.2021.100095 34917926 PMC8669377

[50] Li N Zhou H Wu H Wu Q Duan M Deng W. STING-IRF3 contributes to LPS-induced cardiac dysfunction via NLRP3. Redox Biol. (2019) 24:101215. 10.1016/j.redox.2019.101215 31121492 PMC6529775

[51] Tian M Shi Y Gong X Tan W Guo X Chen Y. MG53 Protects against septic cardiac dysfunction by ubiquitinating ATF2. J Adv Res. (2026) 79:263–75. 10.1016/j.jare.2025.03.031 40107350 PMC12766226

[52] Root-Bernstein R. From co-infections to autoimmune disease via hyperactivated innate immunity: COVID-19 autoimmune coagulopathies, autoimmune myocarditis and multisystem inflammatory syndrome in children. Int J Mol Sci. (2023) 24(3):3001. 10.3390/ijms24033001 36769320 PMC9917907

[53] Root-Bernstein R Huber J Ziehl A Pietrowicz M. SARS-CoV-2 and its bacterial co- or super-infections synergize to trigger COVID-19 autoimmune cardiopathies. Int J Mol Sci. (2023) 24(15):12177. 10.3390/ijms241512177 37569555 PMC10418384

[54] Tungsanga S Udompitsapitak K Worasilchai J Ratana-aneckchai T Wannigama DL Katavetin P. Candida administration in 5/6 nephrectomized mice enhanced fibrosis in internal organs: an impact of lipopolysaccharide and (1 → 3)-β-D-glucan from leaky gut. Int J Mol Sci. (2022) 23(24):15987. 10.3390/ijms232415987 36555628 PMC9784901

[55] Frantz S Ertl G Bauersachs J. Mechanisms of disease: toll-like receptors in cardiovascular disease. Nat Clin Pract Cardiovasc Med. (2007) 4(8):444–54. 10.1038/ncpcardio0938 17653117

[56] Baxan N Papanikolaou A Salles-Crawley I Lota A Chowdhury R Dubois O. Characterization of acute TLR-7 agonist-induced hemorrhagic myocarditis in mice by multiparametric quantitative cardiac magnetic resonance imaging. Dis Model Mech. (2019) 12(8):dmm040725. 10.1242/dmm.040725 31324689 PMC6737951

[57] Xiong J Zhang S Geng Z Lin J Cheng K Hu H. An aberrant resurgence of endogenous retroviruses prompts myocarditis and heart failure. Circulation. (2025) 152(13):939–56. 10.1161/CIRCULATIONAHA.125.074845 40820798 PMC12466171

[58] Dopkins N Nixon DF. Activation of human endogenous retroviruses and its physiological consequences. Nat Rev Mol Cell Biol. (2024) 25(3):212–22. 10.1038/s41580-023-00674-z 37872387

[59] Frantz S Falcão-Pires I Balligand JL Bauersachs J Brutsaert D Ciccarelli M. The innate immune system in chronic cardiomyopathy: a European Society of Cardiology (ESC) scientific statement. Eur J Heart Fail. (2018) 20(3):445–59. 10.1002/ejhf.1138 29333691 PMC5993315

[60] Song EJ Joachimbauer A Tasca S Baylis R Schmidt D Ludewig B. T cells in acute and chronic myocarditis: from diagnosis to treatment. Eur Heart J. (2026) 47(19):2255–70. 10.1093/eurheartj/ehaf1080 41605248 PMC13183180

[61] Chen D Geng Y Deng Z Li P Xue S Xu T. Inhibition of TLR4 alleviates heat stroke-induced cardiomyocyte injury by down-regulating inflammation and ferroptosis. Molecules. (2023) 28(5):2297. 10.3390/molecules28052297 36903542 PMC10005438

[62] Tiller C Reindl M Holzknecht M Lechner I Schwaiger J Brenner C. Association of plasma interleukin-6 with infarct size, reperfusion injury, and adverse remodelling after ST-elevation myocardial infarction. Eur Heart J Acute Cardiovasc Care. (2022) 11(1):113–23. 10.1093/ehjacc/zuab110 34849677

[63] Wang Z Dang Y Li Y Zhang Y Zhou S Zhang Z. Recent advances in ferroptosis-immune inflammation interactions in cardiovascular disease: mechanisms and therapeutic potential. Front Immunol. (2025) 16:1691705. 10.3389/fimmu.2025.1691705 41394800 PMC12695618

[64] Lu RXZ Rafatian N Zhao Y Wagner KT Beroncal EL Li B. Cardiac tissue model of immune-induced dysfunction reveals the role of free mitochondrial DNA and the therapeutic effects of exosomes. Sci Adv. (2024) 10(13):eadk0164. 10.1126/sciadv.adk0164 38536913 PMC10971762

[65] Song J Wang M Li Q Zhao W Chen X Li C. Mangiferin inhibits cGAS-STING pathway-related inflammation via Nrf2 activation. Chin Med. (2026) 21(1):47. 10.1186/s13020-026-01329-9 41572319 PMC12829080

[66] Lai YQ Chong SY Nair V Cui WH Li LJ Liu ST. Toll-like receptor 7 constrains efferocytosis in myocardial injury. Basic Res Cardiol. (2026). 10.1007/s00395-026-01185-6 42141116

[67] Li Y Li X Chen X Sun X Liu X Wang G. Qishen granule inhibits splenic monocytes and protects cardiac function via TLR4-MyD88-NF-*κ*B p65 in heart failure mice. Front Pharmacol. (2022) 13:850187. 10.3389/fphar.2022.850187 35370707 PMC8964526

[68] Tao DD Li Y Tian XJ Liao X-J Yu Z-Q Xiang Z-Y. Effect of FoxO1 on cardiomyocyte apoptosis and inflammation in viral myocarditis via modulation of the TLR4/NF-*κ*B signaling pathway. Int Heart J. (2023) 64(4):732–40. 10.1536/ihj.22-627 37518354

[69] Wu X Li Y Zhang S Zhou X. Ferroptosis as a novel therapeutic target for cardiovascular disease. Theranostics. (2021) 11(7):3052–9. 10.7150/thno.54113 33537073 PMC7847684

[70] Robinson SM Tsueng G Sin J Mangale V Rahawi S McIntyre LL. Coxsackievirus B exits the host cell in shed microvesicles displaying autophagosomal markers. PLoS Pathog. (2014) 10(4):e1004045. 10.1371/journal.ppat.1004045 24722773 PMC3983045

[71] Fan L Tang Q Wang Y Sun H Li G Yang Y. Injury-induced clusterin? Cardiomyocytes suppress inflammation and promote regeneration in neonatal and adult hearts by reprogramming macrophages. Cell Stem Cell. (2025) 32(12):1849–68.e15. 10.1016/j.stem.2025.10.008 41205597

[72] Zhang Y Cui H Zhao M Yu H Xu W Wang Z. Cardiomyocyte-derived small extracellular vesicle-transported let-7b-5p modulates cardiac remodeling via TLR7 signaling pathway. FASEB J. (2024) 38(22):e70196. 10.1096/fj.202302587RRR 39570019

[73] Liew FY Xu D Brint EK O'Neill LAJ. Negative regulation of toll-like receptor-mediated immune responses. Nat Rev Immunol. (2005) 5(6):446–58. 10.1038/nri1630 15928677

[74] Pannucci P Jefferson SR Hampshire J Cooper SL Hill SJ Woolard J. COVID-19-induced myocarditis: pathophysiological roles of ACE2 and toll-like receptors. Int J Mol Sci. (2023) 24(6):5374. 10.3390/ijms24065374 36982447 PMC10049267

[75] Lenz M Kiss A Haider P Salzmann M Brekalo M Krychtiuk KA. Short-term toll-like receptor 9 inhibition leads to left ventricular wall thinning after myocardial infarction. ESC Heart Fail. (2023) 10(4):2375–85. 10.1002/ehf2.14403 37190856 PMC10375131

[76] Chang C Hu L Sun S Song Y Liu S Wang J. Regulatory role of the TLR4/JNK signaling pathway in sepsis-induced myocardial dysfunction. Mol Med Rep. (2021) 23(5):334. 10.3892/mmr.2021.11973 33760172 PMC7974310

[77] Chung CC Kao YH Chen YC Lin Y-K Higa S Hsu K-C. PCSK9 Enhances cardiac fibrogenesis via the activation of toll-like receptor and NLRP3 inflammasome signaling. Int J Mol Sci. (2025) 26(5):1921. 10.3390/ijms26051921 40076547 PMC11900342

[78] Chandler LC Yusuf IH McClements ME Barnard A MacLaren R Xue K. Immunomodulatory effects of hydroxychloroquine and chloroquine in viral infections and their potential application in retinal gene therapy. Int J Mol Sci. (2020) 21(14):4972. 10.3390/ijms21144972 32674481 PMC7404262

[79] Schrezenmeier E Dörner T. Mechanisms of action of hydroxychloroquine and chloroquine: implications for rheumatology. Nat Rev Rheumatol. (2020) 16(3):155–66. 10.1038/s41584-020-0372-x 32034323

[80] Zheng H Zhang Y He J Yang Z Zhang R Li L. Hydroxychloroquine inhibits macrophage activation and attenuates renal fibrosis after ischemia-reperfusion injury. Front Immunol. (2021) 12:645100. 10.3389/fimmu.2021.645100 33936063 PMC8079743

[81] Marsh KM Rastogi R Zhang A Wu D Kron IL Yang Z. Hydroxychloroquine attenuates myocardial ischemic and post-ischemic reperfusion injury by inhibiting the toll-like receptor 9-type I interferon pathway. Cardiol Cardiovasc Med. (2022) 6(4):416–23. 10.26502/fccm.92920278 36081846 PMC9450995

[82] Beetler DJ Giresi P Di Florio DN Fliess JJ McCabe EJ Watkins MM. Therapeutic effects of platelet-derived extracellular vesicles in viral myocarditis. Front Immunol. (2025) 15:1468969. 10.3389/fimmu.2024.1468969 39835120 PMC11743460

[83] Herrera-Martínez AD Hermán-Sánchez N G-García ME Muñoz-Jiménez C Pérez-Gómez JM Montero-Hidalgo AJ. Modulation of inflammasome components in patients with heart failure using oral nutritional supplements: investigating the molecular mechanisms beyond the clinical benefit. Eur J Nutr. (2026) 65(2):39. 10.1007/s00394-025-03878-5 41632188 PMC12868064

[84] Chimenti C Verardo R Scopelliti F Grande C Petrosillo N Piselli P. Myocardial expression of toll-like receptor 4 predicts the response to immunosuppressive therapy in patients with virus-negative chronic inflammatory cardiomyopathy. Eur J Heart Fail. (2017) 19(7):915–25. 10.1002/ejhf.796 28370906

[85] Kouroupis D Zografou I Doukelis P Patoulias D Popovic DS Karakasis P. Presepsin: an emerging biomarker in cardiometabolic disorders. J Pers Med. (2025) 15(4):125. 10.3390/jpm15040125 40278304 PMC12028629

[86] Toprak K Inanır M Memioğlu T Kaplangoray M Palice A Tascanov MB. Could zonulin and presepsin be biomarkers and therapeutic targets for acute myocarditis? Arq Bras Cardiol. (2023) 120:e20230017. 10.36660/abc.20230017 37556677 PMC10464857

[87] Di Florio DN Sin J Coronado MJ Atwal PS Fairweather DL. Sex differences in inflammation, redox biology, mitochondria and autoimmunity. Redox Biol. (2020) 31:101482. 10.1016/j.redox.2020.101482 32197947 PMC7212489

[88] Roberts BJ Dragon JA Moussawi M Huber SA. Sex-specific signaling through toll-like receptors 2 and 4 contributes to survival outcome of coxsackievirus B3 infection in C57Bl/6 mice. Biol Sex Differ. (2012) 3(1):25. 10.1186/2042-6410-3-25 23241283 PMC3586360

[89] Medzhitov R. Origin and physiological roles of inflammation. Nature. (2008) 454(7203):428–35. 10.1038/nature07201 18650913

[90] Hilgendorf I Frantz S Frangogiannis NG. Repair of the infarcted heart: cellular effectors, molecular mechanisms and therapeutic opportunities. Circ Res. (2024) 134(12):1718–51. 10.1161/CIRCRESAHA.124.323658 38843294 PMC11164543

[91] Oduro PK Zheng X Wei J Yang Y Wang Y Zhang H. The cGAS-STING signaling in cardiovascular and metabolic diseases: future novel target option for pharmacotherapy. Acta Pharm Sin B. (2022) 12(1):50–75. 10.1016/j.apsb.2021.05.011 35127372 PMC8799861

[92] Wang BQ Cai XH Li MQ Liu X Xue JH Liu Y. Chaperone-Mediated autophagic degradation of USP9X in macrophages exacerbates postmyocardial infarction inflammation and cardiac dysfunction. Adv Sci (Weinh). (2026) 13(19): e18950. 10.1002/advs.202518950 41604579 PMC13045210

[93] Yang J Yang C Yang J Ding J Li X Yu Q. RP105 Alleviates myocardial ischemia reperfusion injury via inhibiting TLR4/TRIF pathways. Int J Mol Med. (2018) 41(6):3287–95. 10.3892/ijmm.2018.3538 29512709 PMC5881694

